# Novel maternal duplication of 6p22.3-p25.3 with subtelomeric 6p25.3 deletion: new clinical findings and genotype–phenotype correlations

**DOI:** 10.1186/s13039-023-00640-6

**Published:** 2023-06-11

**Authors:** Liyu Zhang, Xiaoling Tie, Fengyu Che, Guoxia Wang, Ying Ge, Benchang Li, Ying Yang

**Affiliations:** 1 Shaanxi Institute for Pediatric Diseases, Xi’an Children’s Hospital, Xi’an, China; 2 Department of Rehabilitation, Xi’an Children’s Hospital, Xi’an, China; 3 The Center Laboratory Medicine, Xi’an Children’s Hospital, Xi’an, China

## Abstract

**Background:**

Copy-number variants (CNVs) drive many neurodevelopmental-related disorders. Although many neurodevelopmental-related CNVs can give rise to widespread phenotypes, it is necessary to identify the major genes contributing to phenotypic presentation. Copy-number variations in chromosome 6, such as independent 6p deletion and 6p duplication, have been reported in several live-born infants and present widespread abnormalities such as intellectual disability, growth deficiency, developmental delay, and multiple dysmorphic facial features. However, a contiguous deletion and duplication in chromosome 6p regions have been reported in only a few cases.

**Case presentation:**

In this study, we reported the first duplication of chromosome band 6p25.3–p22.3 with deletion of 6p25.3 in a pedigree. This is the first case reported involving CNVs in these chromosomal regions. In this pedigree, we reported a 1-year-old boy with maternal 6p25-pter duplication characterized by chromosome karyotype. Further analysis using CNV-seq revealed a 20.88-Mb duplication at 6p25.3-p22.3 associated with a contiguous 0.66-Mb 6p25.3 deletion. Whole exome sequencing confirmed the deletion/duplication and identified no pathogenic or likely pathogenic variants related with the patient´s phenotype. The proband presented abnormal growth, developmental delay, skeletal dysplasia, hearing loss, and dysmorphic facial features. Additionally, he presented recurrent infection after birth. CNV-seq using the proband´s parental samples showed that the deletion/duplication was inherited from the proband´s mother, who exhibited a similar phenotype to the proband. When compared with other cases, this proband and his mother presented a new clinical finding: forearm bone dysplasia. The major candidate genes contributing to recurrent infection, eye development, hearing loss features, neurodevelopmental development, and congenital bone dysplasia were further discussed.

**Conclusions:**

Our results showed a new clinical finding of a contiguous deletion and duplication in chromosome 6p regions and suggested candidate genes associated with phenotypic features, such as *FOXC1*, *SERPINB6*, *NRN1*, *TUBB2A*, *IRF4*, and *RIPK1*.

**Supplementary Information:**

The online version contains supplementary material available at 10.1186/s13039-023-00640-6.

## Background

Neurodevelopmental disorders are the most prevalent and common chronic medical events in paediatrics, with a prevalence of 2–3% in the population around the world [1–3]. In a broader conceptualization, neurodevelopmental disorders include developmental delay (DD), intellectual disability (ID), autism or autism spectrum disorder (ASD), epilepsy, and behavioural abnormalities along with developmental disabilities [4]. Among them, developmental delay (DD) and intellectual disability (ID) are some of the most common conditions in neurodevelopmental disorders, affecting up to approximately 3% of the paediatric population [5–8]. DD and ID are complementary entities separated chronologically because IQ testing is not quite valid and reliable for younger children (younger than 5 years). Therefore, DD is reserved for younger children, and ID is applied for older children [9]. Generally, children’s developmental domains include gross/fine motor development, speech/language development, cognitive development, social/personal development, and activities of daily living. DD/ID is defined as a significant delay in two or more developmental domains. Patients express serious neurodevelopmental disorders during childhood [10].

Neurodevelopmental disorders, especially DD/ID, represent a heterogeneous group of disorders. The reasons for these diseases are quite complicated, including environmental factors and genetic factors. It has been reported that genetic factors contribute at least a quarter to half of the aetiological reasons for DD/ID, including copy-number variants, structural variants, and single-nucleotide variations. To date, copy-number variants comprising chromosomal deletions and duplications have been proven to be a major reason for neurodevelopmental disorders, especially DD and ID [3, 11]. Pathogenic CNVs can alter the structure and function of the genes, resulting in human genetic diseases. The majority of pathogenic CNVs that lose or gain genetic material are based on replication error or DNA repair mechanisms. Approximately 25.7% of children with developmental delay were due to deleterious CNVs larger than 400 kb [12].

Differences in size, the number of affected genes, and even the precise breakpoints of each CNV may cause different neurodevelopmental phenotypes. It has been reported that CNV variations on the short arm of chromosome 6 result in different phenotypes, especially different breakpoints, either interstitial (breakpoints within the 6p22p24 region) or terminal (breakpoints within the 6p24-pter region) [13]. In addition, Martinet et al. reported that the CNV variations in 6p are often more complex, they described an 8.1 Mb 6pter-6p24.3 deletion associated with a contiguous 5.8 Mb 6p24.36p24.1 inverted duplication [14]. Nakane et al. described a patient with a 2.1-Mb 6p25.3 deletion and a 4.14-Mb 6p25.3p25.2 duplication [15]. Other researchers also reported the deletion and duplication on 6p [16–19]. However, the phenotypes of patients are highly heterogeneous. Nevertheless, the 6p deletion associated with a contiguous duplication is rare, and fewer than 10 individuals have been reported [13–15, 17]. To evaluate the variability of clinical features in patients with 6p CNVs, it is necessary to report additional cases to better review genotype–phenotype correlations.

In this study, we reported a patient with neurodevelopmental delay with facial anomalies. His mother exhibited a clinical phenotype that was almost identical to that of the proband. The genomic variations of these pedigree were assessed by chromosome karyotype, whole exome sequencing and copy number variation analysis. The results showed a 6p25.3 terminal deletion associated with a 6p25.3-p22.3 duplication in the proband and his mother. We discuss the phenotype diversity with those of previously reported patients and compared the putative genes in related CNV regions, aiming to establish genotype–phenotype correlations.

## Case presentation

The patient, who is currently a 1.9-year-old Chinese boy, was the first child of nonconsanguineous parents. He is the only child of his parents. He was delivered by caesarean section at 35 weeks and 2 days of gestation due to high blood pressure and hyperglycaemia of his mother. The pregnancy was unremarkable except for unclear nasal bone and imperforate anus noted on ultrasound scan at 28 weeks of gestation. His birth weight was 1400 g (Z score, − 2.85), length was 42 cm (Z score, − 1.83), and occipitofrontal circumference (OFC) was 26 cm (Z score, − 4.19). The neonate's crying manifested a degree of weakness, and the percutaneous oxygenation was inadequate to maintain pace with the infant's spontaneous breathing subsequent to birth. Further examinations showed that he had anal atresia, patent ductus arteriosus, atrial septal defect, and pulmonary hypertension.

**Fig. 1:**
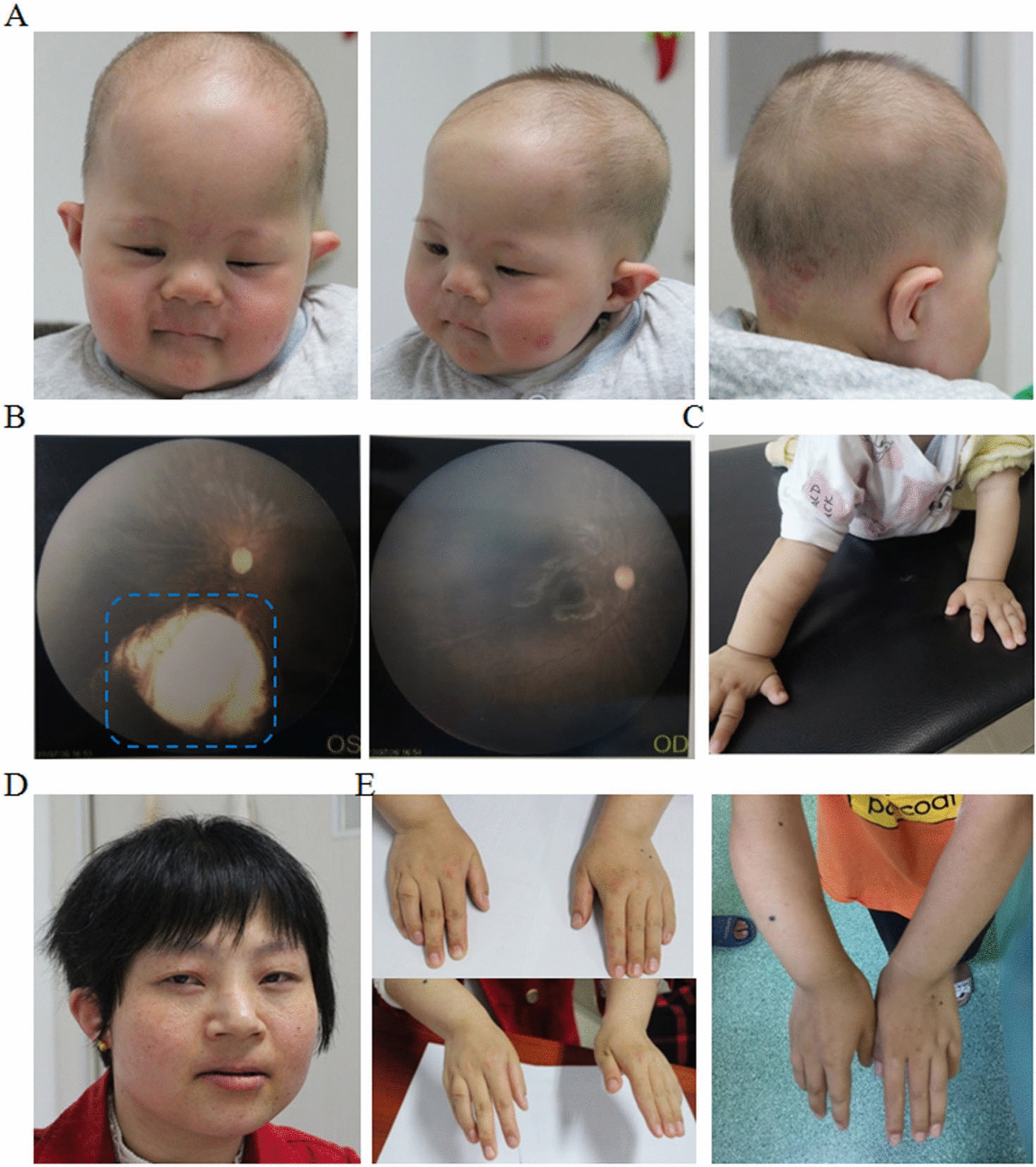
Clinical features of the proband and his mother. **A** Facial appearance of the patient at age 1 year and 5 months; **B** Congenital choroidal defect of the patient (Blue dotted box indicated the choroidal defect); **C** Picture of proband’s forearm. **D** Facial appearance of the mother at age 26 year; **E** Hand and arm picture of the mother

The patient revealed severe feeding difficulties, significant physical growth and neurobehavioural development delay after birth. At 1.4 years old, his weight was 7.73 kg (Z score, − 3.39), length was 65.2 cm (Z score, − 5.04), and occipitofrontal circumference (OFC) was 41.8 cm (Z score, − 4.29). As shown in Fig. 1A, he had multiple dysmorphic facial features, including a higharched palate, long philtrum, thin upper lip, abnormal nasal bridge morphology, upper eyelid entropion, alternating strabismus, nystagmus, and decreased width of the eyelids. His skull shape was abnormal, presenting as bilateral symmetrical defects of the parietal bone. In addition, he had a congenital choroidal defect in the left eye (Fig. 1B). He had congenital heart defects, such as patent foramen ovale, coronary sinus dilation, and persistent left superior chamber. Marked growth impairment was noted. He shows severe psychomotor developmental delay, including motor and intellectual development: at 1.4 years old, he could not sit unsupported, stand unassisted and walk independently. He was uncapable of speaking meaningful words. The Gesell developmental scale test revealed severe global development delay: gross motor skills DQ: 32; fine motor skills DQ: 34; adaptation ability DQ: 25; speaking skills DQ: 28; social capacity DQ: 10.

The results of routine serum biochemical and haematological tests were normal. Brain MRI showed abnormalities in the brain: bilateral ventricles were enlarged asymmetrically. Basic metabolic screening of haematuria, X-ray examination of the hip joint, and posterotemporal CT were also normal. He was found to have profound bilateral sensorineural deafness, with 50 dB in the right ear and 70 dB in the left ear. The patient underwent anal atresia plasty. In addition, although not obvious, the proband showed a slightly bowed radius and ulna. As shown in Fig. 1C, the picture indicated a widened radioulnar joint space and abnormal physiological curvature of the radius (excessive curvature). At present, the patient has been treated by family anal dilatation, and the shape and frequency of stool are basically normal. It should be noted that from birth to now, he had a history of at least five hospitalizations for common pneumonia. All detailed information is presented in Table 1.

**Table 1:** Clinical features of patients with 6p deletions and duplications

Basic information	III 3	II 4	Patient 1	Patient 2	Patient 3	Patient 4	Patient 5	Patient 6	Patient 7
CNV sizes									
Duplication region (hg38)	chr6:820,000–21,699,769	chr6:820,000–21,679,769	chr6:5,159,762–6,167,183	chr6:430,814–6,799,957	chr6:2,331,912–3,032,514	chr6:17,556,755–17,873,607	chr6:13,919,131–15,987,662	chr6:15,181,642–15,490,178	6p24.3-6p24.1
Duplication size (Mb)	20.879	20.86	1.007	6.369	0.701	316.85 kb	2.07 Mb	308.54 kb	5.8
Deletion region (hg38)	chr6:160,000–820,000	chr6:160,000–820,000	chr6:335,924–5,113,947	chr6:182,176–404,928	chr6:1–2,113,525	−	−	−	6pter-6p24.3
Deletion size (Mb)	0.66	0.66	4.778	0.222	2.114	−	−	−	8.1
Sex	Male	Female	Female	Female	Male	Female	Female	Male	Femle
Age at consultation	1y5m	26y	1y1m	New born	4 m				New born
Age at last follow up	1y9m	26y	12y	10y5m	27y	16y	Less than 1 year	11y	NA
Craniofacial features									
Frontal bossing/high forehead	+	−	+	ND	ND	ND	ND	ND	+
Midface retrusion/flat midface	−	−	+	ND	+	ND	ND	ND	+
Hypertelorism/telecanthus	−	−	+	ND	+	+	ND	ND	+
Arched eyebrows	−	−	ND	+	ND	ND	ND	ND	ND
Blepharoptosis	+	−	ND	+	ND	ND	ND	ND	ND
Downslanted palpebral fissures	−	−	ND	ND	ND	ND	ND	ND	+
Sunken eyeballs	+	−	ND	ND	ND	ND	ND	+	ND
External ear anomalies/low-set	+	+	−	ND	ND	ND	ND	ND	+
Wide/depressed nasal bridge	+	−	+	ND	ND	ND	ND	ND	ND
Long/short philtrum	+	+	−	+	ND	+	ND	ND	ND
Smooth/flat philtrum	−	−	ND	+	ND	ND	ND	ND	+
Short/small nose	−	−	ND	+	+	ND	ND	ND	ND
Nasal bone dysplasia	+	−	ND	ND	ND	ND	ND	ND	ND
Flat nose bridge	+	−	ND	+	ND	ND	ND	ND	ND
Thin upper lip	+	+	ND	+	ND	ND	ND	ND	ND
Small mouth	−	−	ND	+	ND	ND	ND	ND	ND
High/cleft palate	−	−	+	ND	+	ND	ND	ND	ND
Cognitive development									
Development delay	+	+	+	+	+	ND	ND	+	+
Intellectual disability	+	+	+	+	+	ND	ND	+	+
Language impairment	+	+	+	+	+	ND	ND	+	+
Hypotonia	+	−	−	+	ND	+	ND	ND	ND
Hearing Loss	+	+	+	ND	ND	ND	ND	ND	+
Heart defect	−	ND	−	+	+	ND	ND	ND	−
Structural eye abnormality									
Glaucoma	−	−	−	+	−	−	ND	ND	ND
Refractive error	−	−	+	−	−	−	ND	ND	ND
Strabismus	−	−	−	−	−	+	ND	ND	+
Corectopia	−	−	+	−	−	−	ND	ND	ND
Far-sightedness	−	−	+	+	−	−	ND	ND	+
Congenital choroidal defect	+	−	−	−	−	−	ND	ND	ND
Corneal opacity	−	−	−	−	+	−	ND	ND	ND
Brain abnormalities									
Encephalatrophy	−	−	ND	ND	+	ND	ND	ND	ND
Skull shape abnormal	−	−	ND	ND	ND	ND	ND	ND	ND
White matter abnormalities	−	−	+	ND	+	ND	ND	ND	ND
Lateral ventricle enlargement	−	−	ND	ND	ND	ND	ND	ND	+
Cerebrospinal fluid fistula	−	−	+	ND	ND	ND	ND	ND	ND
Skeletal anomalies									
Slender long bones and tall	−	−	+	ND	ND	ND	ND	ND	ND
Foreshortened vertebral bodies	−	−	ND	ND	ND	ND	ND	ND	ND
Epiphyseal dysplasia	−	−	+	ND	ND	ND	ND	ND	ND
Hip dysplasia	−	−	−	ND	ND	ND	ND	ND	ND
Hand anomaly	−	+	+	ND	ND	ND	+	ND	ND
Foot anomaly	−	−	+	+	ND	ND	ND	ND	+
Dental abnormalities	−	−	−	ND	ND	ND	ND	ND	ND
Scoliosis	−	−	ND	ND	+	ND	ND	ND	ND
Others									
Nephrotic syndrome	−	−	ND	+	ND	ND	ND	ND	ND
Short stature	+	+	ND	ND	+	ND	ND	ND	ND
Imperforate anus	+	−	ND	ND	ND	ND	ND	ND	ND
Epilepsy	−	−	ND	ND	+	ND	ND	ND	+
Hamartoma	−	+	ND	ND	ND	ND	ND	ND	ND
Recurrent respiratory system Infection	+	ND	ND	ND		ND	ND	ND	ND

**Fig. 2:**
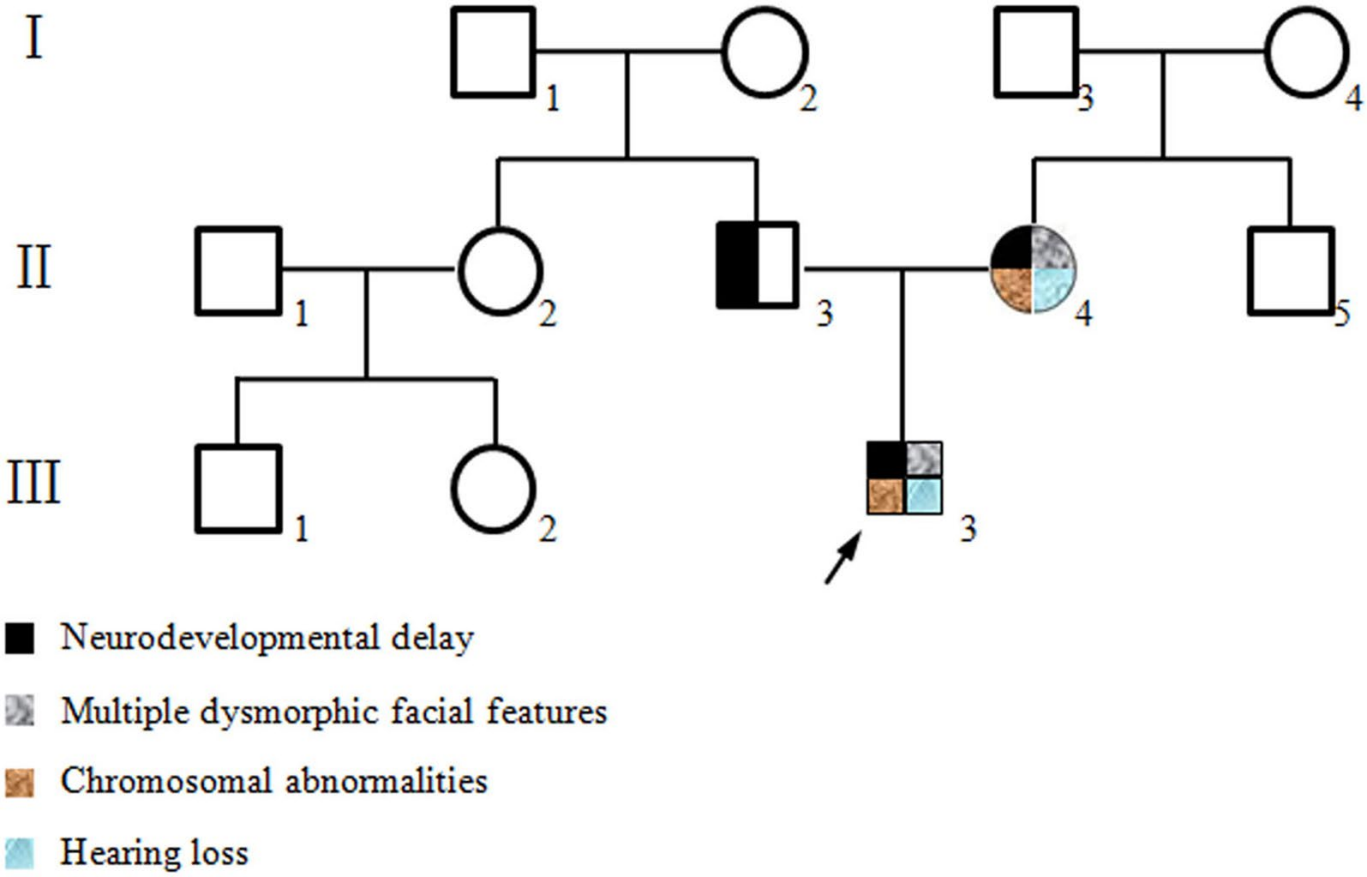
Pedigree of this family

The patient’s mother is a 26-year-old Chinese woman. She was married at 22 years old and was pregnant spontaneously at 25 years old. She is short in stature and 147 cm in height (< P3), and her weight is 41.5 kg (< P10). As shown in Fig. 1D, her facial dysmorphic features are similar to the proband´s features: long philtrum, thin upper lip, and abnormal nasal bridge morphology. She presented developmental delay after 6 months. Additionally, she had congenital moderate intellectual disability. She was uncapable of communicating with others normally and could hardly understand conversations now. Ultrasonic testing found that there was a hamartoma in her left kidney. In addition, her arms began to bend after birth, and now her arm and fingers could not be fully extended due to joint contractures (Fig. 1E), which was quite similar to the proband. The patient’s father was normal and developed well after birth. After a history of Japanese encephalitis at 3 years old, he had difficulty speaking, and neurocognitive development was impaired mildly. The pedigree of this family is shown in Fig. 2.

**Fig. 3:**
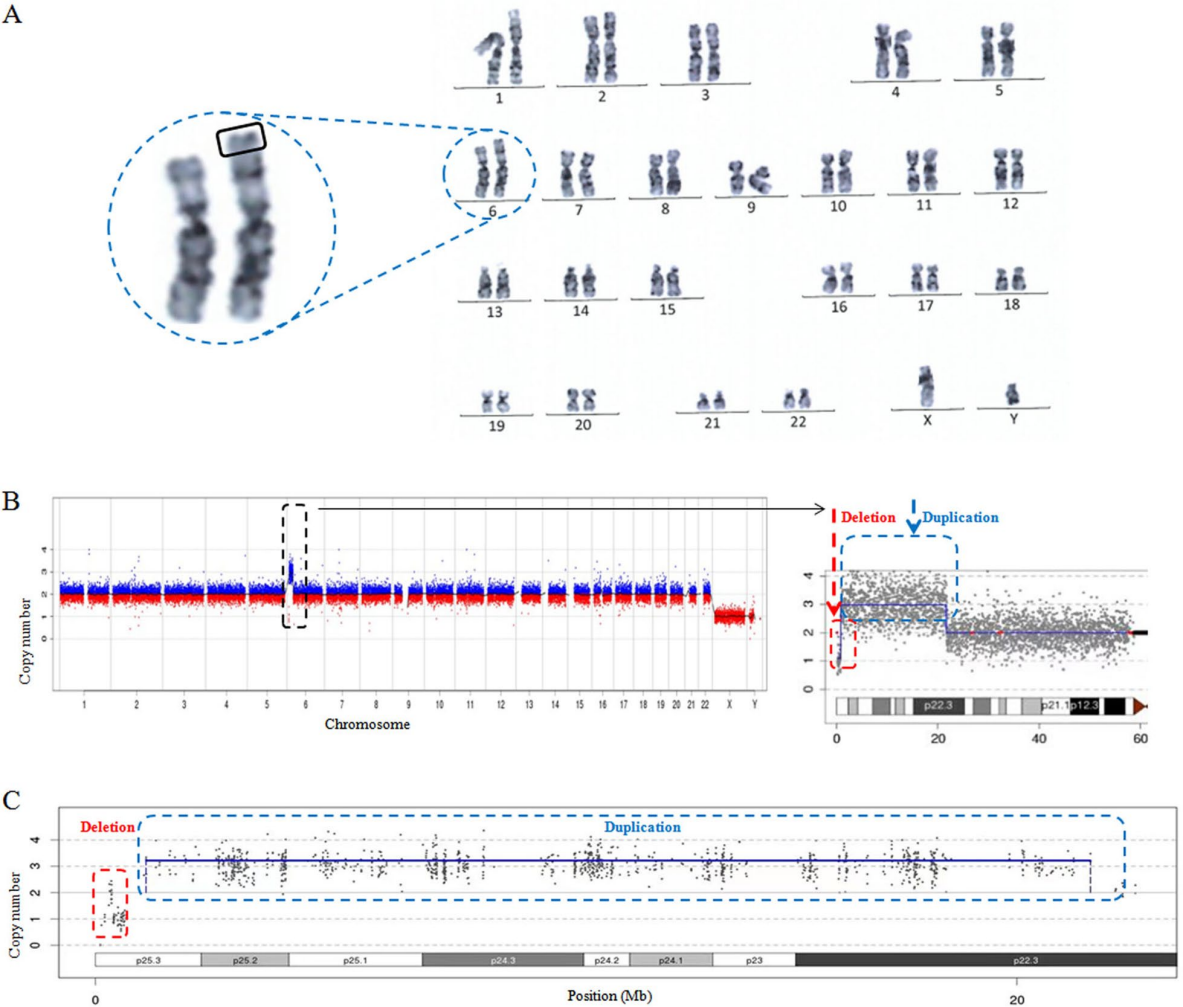
Genetic analysis of the proband. **A** Chromosome karyotype; **B** CNV-seq result showed a 20.88 Mb heterozygous duplication at 6p25.3-p22.3 and a 0.66 Mb heterozygous deletion at 6p25.3; **C** WES-CNV revealeda 0.3-Mb heterozygous deletion at 6p25.3 and a 20.49-Mb heterozygous duplication at 6p25.3-p22.3

To determine the cytogenetic and molecular variants of the proband, whether there were any chromosome abnormalities in the proband was evaluated, and chromosome analysis was utilized first. Two millilitres of peripheral blood from the patient was exsanguinated in a heparin anticoagulant tube. The blood sample was cultured with RPMI 1640 cell culture media, and 3–5 drops of colchicines (50 mg/mL) were added to the cells and further incubated for 1 h. Then, the cells were collected and incubated with 8 mL KCl buffer (0.075 mol/L) for 25 min. The cells were fixed and stained with Giemsa solution. Next, the cells were observed under a microscope, and the chromosome karyotype was analysed. Genomic DNA and metaphase chromosomes were obtained from the peripheral blood leukocytes of the proband. Chromosome analysis showed 46, XY, add(6)(p25), indicating a suspicious addition in chromosome 6 for unknown reason (Fig. 3A). No apparent pathogenic copy number variations were found in other chromosomal regions.

To further detect the suspicious addition in chromosome 6, CNV-seq was applied for the proband. DNA was extracted from trio samples by MagMAX DNA Ultra 2.0 (Thermo Fisher, CA, USA). DNA samples were sequenced on the NGS platform (Berry Genomics, Beijing, China). A PCR-free-frag library was constructed for CNV-seq. Approximately 5 million 37-bp plus 8-bp (index) raw reads were generated for each sample after library sequencing on the NextSeq CN500 platform (Berry Genomics). Reads were processed, and CNVs were evaluated by an in-house pipeline using read counts based on a smoothness model (Berry Genomics, Beijing, China) according to the previous description. Surprisingly, the CNV-seq results not only showed a 20.88-Mb heterozygous duplication at 6p25.3-p22.3 (chr6:820,000–21,699,769, hg38) but also a 0.66-Mb heterozygous deletion at 6p25.3 (chr6:g.160000–820,000, hg38) (Fig. 3B).

The 20.88-Mb heterozygous duplication at 6p25.3p22.3 (chr6: 820,000–21,699,769, hg38) has not been recorded in the Database of Genomic Variants. This duplication region contains 89 RefSeq protein-coding genes, including *FOXC1*, *TUBB2B*, and *NRN1*. The upstream duplication breakpoint and downstream duplication breakpoint are in an intergenic region. The Decipher database and literature have reported several cases whose duplication regions overlapped or contained the proband’s duplication region. Almost all of these cases presented underdevelopment and multiple dysmorphic features. The Clingen CNV comprehensive score was greater than 0.99 (1.5). Therefore, this duplication region was classified as pathogenic CNV according to the ACMG rating guidelines for CNV [20].

The 0.66-Mb heterozygous deletion at 6p25.3 (chr6:g.160000–820,000, hg38) has not been recorded in the Database of Genomic Variants either. This deletion region contains 4 RefSeq protein-coding genes, including *DUSP22, EXOC2, HUS1B* and *IRF4*. Cases with deletion regions overlapping this deletion have rarely been reported, and only one male patient whose 6p25.2 presented a 0.72-Mb paternal deletion region was recorded in the Decipher database (Decipher#280,500). This patient presented with global developmental delay. The Clingen CNV comprehensive score was between − 0.89 and 0.89 (0). Therefore, this deletion region was classified as an uncertain significance CNV according to the ACMG rating guidelines for CNV.

To further exclude the possibility of other single-gene diseases, whole exome sequencing of the proband was performed. Whole exome sequencing results were compared by Sprinkl for CNV calling, and copy number calculation and CNV identification were performed in exons and long segment areas. The genomic coordinates of the WES results are indicated according to NCBI build 38 (hg38). After bioinformatics analysis, no pathogenic or likely pathogenic mutations related to phenotype were found. However, WES-CNV results revealed a 0.3-Mb heterozygous deletion at 6p25.3 (chr6:391,751–693,084, hg38) and a 20.49-Mb heterozygous duplication at 6p25.3-p22.3 (chr6:1,101,132–21,595,959, hg38) (Fig. 3C). Taking the CNV region and WES probe location together, the upstream breakpoint should be between chr6:1,053,627 and chr6:1,101,132, and the downstream breakpoint should be between chr6:21,595,959 and chr6:22,136,495.

To further verify the CNV parental origin of the proband, CNV-seq analysis was utilized for the proband’s mother and father. The CNV-seq result of the proband’s father revealed no pathogenic deletion or duplication variations (Additional file 1: Fig. S1A). The CNV-seq results of the mother indicated the presence of the same deletion/duplication that was identified in the proband. (Additional file 1: Fig. S1B). In addition, the chromosome karyotype of the mother showed 46, XY, add(6)(p25) (Additional file 1: Fig. S1C).

## Discussion and conclusions

In this study, we report a genetic analysis-based thorough investigation of a patient with contiguous deletion and duplication in chromosome 6, manifesting with global developmental delay. G-banded chromosomal karyotype and CNV-seq findings, as well as WES-CNV, would be compatible with a duplication of 6p22.3-p25.3 complicated by a distal 6p25.3 deletion. The patient’s mother presented a similar phenotype, and chromosome abnormalities were consistent with her son, indicating maternal inheritance.

We noticed that there were slight differences between the phenotypes of the proband and his mother. First, the eye abnormalities of the proband were more severe than those of his mother. The proband presented upper eyelid entropion, alternating strabismus, and nystagmus. His mother showed upper eyelid entropion but without alternating strabismus and nystagmus. We speculated that this may be related to environmental factors, other unknown pathogenic genes or some regulatory factors. Second, we found some differences in the clinical symptom severity of the arm and hands. At present, we have only observed slight bend growth in the proband, but his mother presented obvious bent long bone and even joint contractures in the hands’ fingers. Her arm and fingers could not be fully extended. After investigating her medical history, it should be noted that her radius and ulna began to bend grow at a very young age and gradually developed more seriously. Therefore, we should indicate that these differences may be due to age of onset. Finally, the proband presented with recurrent upper and lower respiratory tract infections. However, his mother did not show similar recurrent infections at present. Due to the unavailability of adequate information from his mother's parents and her current inability to recall and recount her medical history from a younger age, we were unable to ascertain whether she had experienced recurrent infections during her childhood. Therefore, it is difficult to compare the symptoms of the proband and his mother at the same age. With regard to the proband’s repeated infection, but his mother is not as an adult, we speculated that the proband’s recurrent infection may be associated with the following reason: when compared with adults, children’s immune cells have not developed memory or antigen specificity for most of the antigens, which may result in infections. Therefore, infections are always more severe at younger ages and may be greatly eased with increasing age.

### Duplication region difference between the proband and his mother

Our proband and his mother presented quite similar phenotypes. Utilizing CNV-seq, the proband showed a 20.88-Mb heterozygous duplication at 6p25.3-p22.3 (chr6:820,000–21,699,769, hg38) and a 0.66-Mb heterozygous deletion at 6p25.3 (chr6:g.160000–820,000, hg38). The results of the mother showed a 0.66-Mb heterozygous deletion at 6p25.3 (chr6:g.160000–820,000, hg38) and a 20.86-Mb heterozygous duplication at 6p25.3-p22.3 (chr6:820,000–21,699,769, hg38). Therefore, it could be assumed that the deletion and duplication in chr6 of the proband is inherited from the mother.

However, it should be noted that the duplication between the proband and his mother exhibited subtle differences, and the variation region of the mother was 20 kb smaller than that of the proband. We assumed that this difference was due to the technical restriction of CNV-seq. CNV-seq is a low-depth whole genome sequencing method with an average sequencing depth of 0.1× . The processed reads were divided into contiguous 20 kb bins. In other words, CNV variation differences under 20 kb were credible.

In addition, we noticed that the CNVs detected via WES-CNV and CNV-seq had some discrepancies: CNVs detected via CNV-seq were larger than those detected via WES. This may be due to the method differences between WES-CNV and CNV-seq. WES-CNV is better capable of assessing the breakpoints in exonic regions, even deletions or duplications in a single exon. However, WES-CNV could not detect regions beyond the exons. When compared, although the limited resolution of CNV-seq is not allowed to detect deletion or duplication regions smaller than 100 kb, CNV-seq can not only be evaluated at the genome level for contiguous deletions or duplications in exonic regions but also in intron regions [21–23]. Therefore, the different advantages and limitations of WES-CNV and CNV-seq are the major reason why the CNVs detected via WES-CNV and CNV-seq have discrepancies.

### Genotype and phenotype comparison

**Fig. 4:**
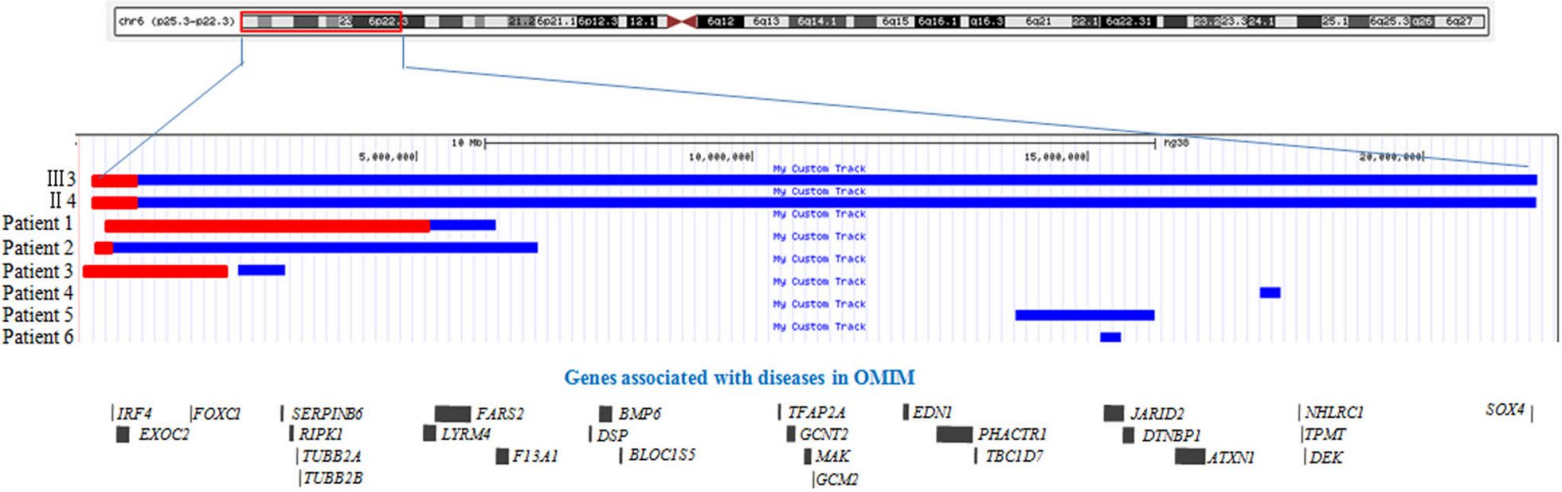
A contiguous deletion and duplication in chromosome 6p regions of our patient and previously reported patients. Red indicated deletion and blue indicated duplication

To date, as shown in Fig. 4 and Table 1, at least 4 patients with contiguous duplication and deletion in chromosome 6 have been reported to have multiple clinical phenotypes. When compared with these cases, the duplication region of our proband was larger, and the deletion region was smaller.

We noticed that all these patients, including our patients, had variable deletions in the 6p25-pter [24]. Submicroscopic deletions of 6p, especially at 6p25 to 6pter, have been reported in several studies and could lead to hearing impairment, ocular dysgenesis, and brain abnormalities. In addition, most patients show dysmorphic features, such as a low nasal bridge and downslanting palpebral fissures. Other features, such as skeletal, renal, and cardiac malformations, are variable [25, 26]. We have noticed that the clinical phenotype and severity of all previously reported 6p25 cytogenetic abnormalities are dependent on altered doses of a gene or genes in the duplicated or deleted regions (Tables 2 and 3). Our proband and his mother have main physical findings: eye abnormalities, developmental delay, language impairment, intellectual disability, growth retardation, and dysmorphic features [27]. The Database of Genomic Variants reported several duplications in the 6p25-p22 region, but they were much smaller. We assumed that when no triple-dosage sensitive gene exists, chromosomal deletions may cause more severe and more serious clinical effects than duplications.

**Table 2:** RefSeq protein-coding genes in a 20.88-Mb Duplicated Region at 6p25.3-p22.3 in the present patient

Gene symbol	OMIM ID	Associated diseases	Inheritance
*ATXN1*	601,556	Spinocerebellar ataxia 1	AD
*BLOC1S5*	607,289	Hermansky-Pudlak syndrome 11	AR
*DSP*	125,647	Arrhythmogenic right ventricular dysplasia 8	AD
		Cardiomyopathy, dilated, with woolly hair and keratoderma	AR
		Dilated cardiomyopathy with woolly hair, keratoderma, and tooth agenesis	AD
		Epidermolysisbullosa, lethal acantholytic	AR
		Keratosis palmoplantarisstriata II	AD
		Skin fragility-woolly hair syndrome	
*DTNBP1*	607,145	Hermansky-Pudlak syndrome 7	AR
*EDN1*	131,240	Auriculocondylar syndrome 3	AR
		Question mark ears, isolated	AD
		{High density lipoprotein cholesterol level QTL 7}	
*F13A1*	134,570	Factor XIIIA deficiency	AR
		{Myocardial infarction, protection against}	–
		{Venous thrombosis, protection against}	AD
*FARS2*	611,592	Combined oxidative phosphorylation deficiency 14	AR
		Spastic paraplegia 77, autosomal recessive	AR
*FOXC1*	601,090	Anterior segment dysgenesis 3, multiple subtypes	AD
		Axenfeld-Rieger syndrome, type 3	AD
*GCM2*	603,716	Hyperparathyroidism 4	AD
		Hypoparathyroidism, familial isolated 2	AD, AR
*GCNT2*	600,429	Adult i phenotype without cataract	AD
		Cataract 13 with adult i phenotype	AR
		[Blood group, Ii]	AD
*LYRM4*	613,311	?Combined oxidative phosphorylation deficiency 19	AR
*MAK*	154,235	Retinitis pigmentosa 62	AR
*NHLRC1*	608,072	Epilepsy, progressive myoclonic 2B (Lafora)	AR
*NQO2*	160,998	{?Breast cancer susceptibility}	AD, Smu
*PHACTR1*	608,723	Developmental and epileptic encephalopathy 70	AD
*RIPK1*	603,453	Autoinflammation with episodic fever and lymphadenopathy	AD
		Immunodeficiency 57 with autoinflammation	AR
*SERPINB6*	173,321	?Deafness, autosomal recessive 91	AR
*SOX4*	184,430	Coffin-Siris syndrome 10	AD
*TBC1D7*	612,655	Macrocephaly/megalencephaly syndrome, autosomal recessive	AR
*TFAP2A*	107,580	Branchiooculofacial syndrome	AD
*TPMT*	187,680	{Thiopurines, poor metabolism of, 1}	AR
*TUBB2A*	615,101	Cortical dysplasia, complex, with other brain malformations 5	AD
*TUBB2B*	612,850	Cortical dysplasia, complex, with other brain malformations 7	AD

**Table 3:** RefSeq protein-coding genes in a 0.66-Mb deleted region at 6p25.3 in the present patient

Gene symbol	OMIM ID	Associated diseases	Inheritance
*IRF4*	601,900	[Skin/hair/eye pigmentation, variation in, 8]	–
*EXOC2*	615,329	Neurodevelopmental disorder with dysmorphicfacies and cerebellar hypoplasia	AR

Linhares et al. reported a girl who exhibited 6p25.3p25.1 terminal deletion associated with a 6p25.1 duplication (Patient 1 in Table 1) [13]. Our patient (proband and his mother) showed similar clinical features to this patient, including dysmorphic features (frontal bossing, depressed nasal bridge), eye problems (strabismus), hearing loss, developmental delay, and intellectual disability. In addition, this patient also presented some overlapping phenotypes with our patients, such as dysmorphic features (severe hypertelorism, midface hypoplasia, high palate) and skeletal abnormalities (total absence of carpal bones and epiphysis ossification, slender long bones, tall vertebral bodies). It should be noted that this patient showed extra phenotypes, such as brain abnormalities (dilated brain ventricles, cerebellar hypoplasia, rotation of the vermis away from the brainstem, highlighted leukopathy), eye abnormalities (strabismus and anterior eye), and seizures. We assumed that these phenotypes may be due to multiple genes in a larger deletion region in 6p25.3. Her deletion region was 4.778 Mb ranging from chr6:335,924 to chr6:5,113,947. This deletion contains eye development-associated genes (*FOXC1*) [28–30] and neurodevelopmental disorder- and seizure-associated genes (*GMDS*, *TUBB2A*, *TUBB2B*) [31–37]. Therefore, we assumed that these factors mainly contributed to these extra phenotypes.

Patient 2 was a Japanese girl reported with a 6.4-Mb duplication at 6p25.3-p25.1 and a 220-kb deletion at 6p25.3 [17]. When compared, we found that the clinical features between patient 2 and our proband were more similar than those of patient 1 [13]. We surmise that the reason is that the duplication and deletion regions of patient 2 are more similar than those of patient 1. Patient 2 and our proband showed similar facial features, such as a short nose with a flat nasal root, hypoplastic alaenasi, a long and flat philtrum, and a thin upper lip vermilion. In addition, motor, intellectual and language development were all delayed. However, except for these similar symptoms, patient 2 showed obvious cardiovascular system abnormalities (ventricular septal defects, patent ductus arteriosus, and tetralogy of Fallot), seizures, and renal problems. She was diagnosed with nephrotic syndrome at 2 years old. Despite extensive treatment, her renal situation worsened. She underwent renal transplantation at nine years old. It should be noted that patient 2, with both smaller deletion and duplication regions, presented severe symptoms. We found that the duplication in our proband is tandem, and patient 2 would be compatible with an inverted duplication of 6p25.3–p25.1 complicated by a distal 6p25.3 deletion. This structural variation may contribute to the differences between patient 2 and the proband. To confirm this, the breakpoint of patient 2 should be analysed to check whether there exists a fusion gene or whether gene transcription regulation is affected. Another reason may be that the age of our proband is younger than the age at onset of renal symptoms; thus, long-term follow-up is needed to observe whether the proband develops similar symptoms.

Patient 3 and patient 7 (Table 1) both had a deletion and duplication in 6p, which may be due to a typical “inv dup del” pattern derived from U-type exchange [14, 15]. They showed similar phenotypes to our patients, such as facial abnormalities, eye abnormalities (iris hypoplasia and congenital glaucoma), and epilepsy. The deletion regions of patients 3 and 7 included the *FOXC1* gene, which has been indicated that its haploinsufficiency can be responsible for glaucoma.

Patients with 6p duplications reported in the literature or database often show intrauterine growth retardation, short stature, microcephaly, prominent forehead, and others, as shown in patient 4 (Decipher#402,407), patient 5 (Decipher#282,057) and patient 6 (Decipher#345,282), which could explain the prominent forehead and short stature of these family patients. In addition, we found that some clinical features could not be explained by 6p deletion or 6p duplication, including recurrent respiratory system infection and imperforate anus. Therefore, the genotype–phenotype correlation in our proband should be discussed further.

### Genotype–Phenotype correlation

#### Gene associated with eye phenotype

Our patient presented with a congenital choroidal defect and an eye phenotype. The eye phenotype is commonly observed in 6p deletion patients, including glaucoma, refractive error, strabismus, corectopia, far-sightedness, and corneal opacity. Several reports have suggested that the *FOXC1* gene may be the major contributor to the eye phenotype of patients with 6p25 deletion syndromes. The *FOXC1* gene is a member of the forkhead family of transcription factors [38].

It has been validated that the *FOXC1* gene is haploinsufficient and that deletion or pathogenic variations of the *FOXC1* gene could cause a variety of anterior eye chamber abnormalities associated with glaucoma, including Axenfeld-Rieger syndrome (OMIM*602,482) and anterior segment dysgenesis 3, multiple subtypes (OMIM*601,631) [39–43]. Patients 1 and 3 in Fig. 4 and Table 1 with deletion of the *FOXC1* gene presented refractive error, corectopia, far-sightedness, and corneal opacity, which were consistent with previous reports.

Our proband with *FOXC1* gene duplication also showed an eye phenotype. Unlike Patients 1 and 3, the proband had mainly congenital choroidal defects. Interestingly, patient 2, presenting far-sightedness, also had a *FOXC1* gene duplication. Nishimura et al. analysed the *FOXC1* gene in 70 probands with congenital anterior chamber defects [39]. Among them, 2 patients with iris hypoplasia or Peters anomaly had duplications of 6p25, which encompasses the *FOXC1* gene. The authors suggested that both deletion and duplication of *FOXC1* may cause anterior chamber defects of the eye. Therefore, we have reasons to assume that *FOXC1* gene duplication contributes majorly to eye symptoms.

It should be noted that although we emphasized the function of the *FOXC1* gene in eye symptoms here, *FOXC1* is currently the most recognized crucial causative gene for 6pter-p24 deletion syndrome, which is involved in a wide range of biological functions and may be associated with abnormalities of multiple systems in patients. Researchers have found that the *FOXC1* gene also plays an important role in cardiac, craniofacial, auditory and cerebellar development. These will be discussed in the following discussion section.

#### Gene associated with hearing loss

Hearing loss could be observed in both 6p deletion syndrome and 6p duplication syndrome. The *SERPINB6* gene (OMIM*173,321) is a kind of serpin peptidase inhibitor. The *SERPINB6* gene was first defined as a hearing loss-associated gene in a consanguineous Turkish family in 2010[44, 45]. All affected members presented nonsyndromic sensorineural hearing loss. Sirmaci et al. found that SERPINB6 protein was expressed in hair cells [45]. Tan et al. found that after homozygous replacement of *SERPINB6A* (the orthologue of human *SERPINB6*) in mice, they showed progressive hearing loss concomitant with cochlear degeneration after 2 weeks of age [46]. The effect appeared developmentally from outer hair cells, inner hair cells, primary auditory neurons, and fibrocytes. The *SERPINB6* gene is located at 6p25.3 and was duplicated in our proband [47]. Current cases with hearing loss reported were mostly *SERPINB6* gene deletions. Remarkably, the *SERPINB6* gene of patient 3 in Fig. 4 and Table 1 was also duplicated, and he also showed hearing loss. Therefore, we have reasons to assume that alterations in another gene in CNV regions may contribute to hearing loss and that the *SERPINB6* gene may cause hearing loss.

The *FOXC1* gene, as mentioned above, is essential for development. The *FOXC1* gene can affect the maintenance of many kinds of cells, such as haematopoietic stem cells, progenitor cell maintenance, and hair follicle stem cells [48, 49]. Scientific reports found that the *FOXC1* gene could lead to hearing loss. Mears et al. reported a patient who carried a heterozygous mutation in the *FOXC1* gene and showed deafness [50]. In addition, several papers have described patients with hearing loss and developmental delay, and further analysis confirmed the deletion of *FOXC1* at 6p25 [51–54]. Therefore, we could not rule out the effect of *FOXC1* on hearing loss in our patients.

#### Gene associated with neurodevelopmental phenotype

*NRN1* gene (OMIM*607,409), which encodes neuritin-1, is a glutamate and neurotrophin receptor target gene. Neuritin is a GPI-anchored protein and can promote neurite outgrowth, which is crucial for the branching of neuritic processes in primary hippocampal and cortical cells [55]. Many reports associated with brain NMR abnormalities have been performed in 6p25 deletion syndrome, which contains the *NRN1* gene. Brain NMR abnormalities, including Dandy-Walker and white matter abnormalities, include brain leukopathy, dilated brain ventricles, cerebellar hypoplasia, short/thin corpus callosum, small cerebellar vermis and dilated fourth ventricle [56]. Several researchers have stressed that the *NRN1* gene should be a major gene for neurodevelopment of the patient features. The *NRN1* gene may be critically associated with neurobiology and influence cognitive dysfunction [57–59]. In our pedigree, both the proband and his mother presented intellectual disabilities, which belong to the neurodevelopmental phenotype. Linhares ND et al. reported that a patient with *NRN1* gene duplication also showed neurodevelopmental delay [13]. Although there are situations in which haploinsufficiency and increased gene dosage may cause the same phenotype, we still suggest that the *NRN1* gene may be associated with the neurocognitive phenotype.

In addition, as we mentioned before, in addition to *NRN1*, we still suspect the function of the *FOXC1* gene in neurodevelopment. Maclean et al. reported several patients with *FOXC1* variations, these patients had mild to moderate developmental delay, and magnetic resonance imaging showed that they had CNS anomalies such as hydrocephalus and hypoplasia of the cerebellum, brainstem, and corpus callosum [51]. Although the majority of probands detected had *FOXC1* de novo mutations or deletions, Nishimura et al. reported 70 probands with congenital anterior chamber defects and other phenotypes, such as neurodevelopmental delay, and members from 2 families encompassed the duplication of the *FOXC1* gene [39]. These results indicated that not only *FOXC1* haploinsufficiency but also increased *FOXC1* gene dosage may cause phenotypes. Therefore, we speculated that the *FOXC1* gene may contribute to the neurodevelopmental phenotype.

The *TUBB2A* gene [OMIM*615101], encoding tubulins, is significant for microtubules and functions in mitosis, intracellular transport, neuron morphology, and ciliary and flagellar motility. Currently, it has been validated that *TUBB2A* gene variations could lead to cortical dysplasia, complex, and other brain malformations 5 [OMIM*615763], and patients may present severe neurodevelopmental disorders, such as diffuse simplified gyral patterns in the brain, enlarged ventricles, and mildly enlarged posterior fossa. In addition, seizures and global developmental delay are also common [34, 60]. Although reported cases carried *TUBB2A* deletion, indicating that the specific developmental brain malformations and neurodevelopmental phenotype were due to *TUBB2A* haploinsufficiency, cases in our pedigree and patient 2 in Table 1 with *TUBB2A* duplication also presented neurodevelopmental delay. Therefore, we believe that the *TUBB2A* gene may also contribute to the neurodevelopmental phenotype.

#### Gene associated with skeletal phenotype

In this pedigree, we found a new clinical finding in the proband and his mother when compared with other cases: forearm bone dysplasia. Both the proband and his mother showed a bowed radius and ulna, which have not been reported in the literature. After analysing the genes involved, we indicated that the *TUBB2A* gene may be essential for skeletal development.

The *TUBB2A* gene [OMIM*615101], as we mentioned above, is related to neurodevelopment. Additionally, we indicated that the *TUBB2A* gene may also be associated with the skeletal phenotype. Although there have been no reports indicating the relationship between duplication of the *TUBB2A* gene and skeletal phenotype, after enquiring the MINT database, Linhares et al. found that the TUBB2A protein could interact with CUL7 [OMIM*609577], which could lead to 3-M syndrome [OMIM*273750] [13]. 3-M syndrome is an autosomal recessive disorder characterized by main skeletal anomalies. The patients showed long and slender tubular bones, delayed bone age, and other skeletal manifestations [61]. Although there has been no direct evidence demonstrating the function of the *TUBB2A* gene in skeletal development, the interaction between TUBB2A and CUL7 suggests that *TUBB2A* may be involved in the skeletal phenotype after there are no pathogenic mutations in the known genes related to skeletal features.

#### Gene associated with recurrent infection

The proband in this pedigree showed recurrent infections, including upper respiratory tract infection and lower respiratory tract infection (pneumonia). He has been hospitalized at least eight times for recurrent respiratory infections since birth. After analysing the genes involved in deletion and duplication regions, although several genes may contribute, we assumed that the *IRF4* gene (OMIM*601,900) may be the major gene for recurrent infection [62]. The *IRF4* gene was first reported to be associated with skin/hair/eye pigmentation (OMIM*611,724) in the OMIM database [63, 64]. The single-nucleotide polymorphisms in the *IRF4* gene have the strongest association with hair colour, skin colour, eye colour, and skin tanning response to sunlight [65]. In addition, researchers have found that the *IRF4* gene is essential for the development of T helper-2 (Th2) cells, Th17 cells, and Th9 cells, and it is interferon regulatory factor 4 [66].

Although the *IRF4* gene has not been found to be associated with some kind of immunodeficiency and the inheritance is unclear, it has been confirmed that the *IRF4* gene plays a role in immunity [62, 67]. The fusion gene with *IRF4* or mutation could contribute to an aberrant IRF4 regulatory network and further fuse the gene expression programs of normal plasma cells and activated B cells [66, 68]. Yu et al. reported six case reports of large B-cell lymphoma with *IRF4* rearrangement [69]. Benatti S reported that the *IRF4* mutation (L116R) could promote the proliferation of chronic lymphocytic leukaemia B cells [70]. It should be noted that Bravo García-Morato M reported a 5-month-old Spanish girl to nonconsanguineous parents. The girl presented primary immune deficiencies (PIDs), including bronchopneumonia and long periods of fever without focus. After genetic analysis, they found that the combined PID was caused by a homozygous splicing mutation in *IRF4* (NM_001195286:c.1213-2A > G,p.V405GfsTer127) [71].

Mittrucker et al. created IRF4 protein-deficient mice by knocking out exons 2 and 3 [72]. They found that mutant mice had poor T- and B-lymphocyte proliferative responses and lacked production of all serum Ig subclasses. Therefore, IRF4 is essential for T- and B-lymphocyte function. Ochiai et al. generated mixed bone marrow chimaeras with mouse IRF4^+/+^ and IRF4^−/−^ progenitors and proposed a model of kinetic control in which signalling-induced dynamics of IRF4 in activated B cells controls their cell-fate outcomes: IRF4 could bind with interferon sequence response elements and further enrich for genes involved in plasma cell differentiation [73]. Staudenraus D et al. mentioned that the point mutation L116R in *IRF4* differentially impacts key cytokine production in Th2, Th9, and Th17 cells [74]. Cook SL et al. reported that *IRF4* haploinsufficiency may impair the affinity maturation of B cells [75].

In summary, although there are few case reports of recurrent infection due to *IRF4* deletion, *IRF4* is essential for immune cells, and *IRF4* deletion may contribute to immune system problems such as recurrent infections.

Beside *IRF4* gene, *RIPK1* gene [OMIM: 603453] was also suspected to be responsible for recurrent infection. *RIPK1* gene encodes a cytosolic kinase and could control multiple signaling pathways leading to inflammation and apoptotic or necroptotic cell death. It has been reported that RIPK1 is an essential molecule in programmed necrosis pathway, which is crucial for immunity, development, and tissue response [76]. At present, *RIPK1* gene has been reported to be associated with two disease: autoinflammation with episodic fever and lymphadenopathy (AIEFL, OMIM: 618,852, AD) and immunodeficiency 57 with autoinflammation (OMIM: 618,108, AR). Although there has no report about *RIPK1* gene duplication leading to disease, Tao et al., have reported two AIEFL patients with heterozygous *RIPK1* gene mutation D324H and D324V via exome sequencing and further confirmed by Sanger sequencing. When compared with control group, the patients serum presented higher level of inflammatory cytokines and chemokines like IL-6, TNF, and gamma-IFN. In addition, the patients’ immune cells showed excessive inflammatory response and activated MAPK signalling pathway, and increased sensitivity could be reversed by inhibiting RIPK1. Meanwhile, in vitro studies utilizing patient fibroblasts indicated the mutation could block the RIPK1 cleavage by caspase-8. Taken together, it should be suspected that these mutations could result in a gain-of-function effect, and further influence inflammatory response [77]. In our patient, *RIPK1* gene was duplicated. although *RIPK1* gene has not been valiadated as triplosensitive gene, it is still considered as a candidate gene contributing to the recurrent infection of our proband.

#### Gene associated with renal abnormalities

Patients with 6p duplications mostly presented renal complications such as protein uria, renal failure, hypoplastic/aplastic kidney, hydronephrosis, ectopic kidney, horseshoe kidney, et al. The variation of the renal complications in 6p duplication syndrome would be compatible with congenital anomalies of the kidney and urinary tract (CAKUT) [17, 78–80], but no genes was localized within CNV variations of patient 2. Finally, *FOXC1* gene was suspected to have some etiological contribution in renal abnormalities: (i) several literatures reported that *FOXC1* gene mutation could cause renal dysplasia, duplex kidney and double ureters in mice [81, 82]; (ii) Patients with renal abnormalities carried a 3-bp (GGC) insertion variation in the six GGC repeats in the coding region of *FOXC1* were reported [83]. However, it is not clear this in-frame insertion variation is causal for these patients.

Patient 2 presented renal abnormalities and *FOXC1* gene was duplicated. When compared, as shown in Fig. 4, *FOXC1* gene duplication was also seen in our proband (III3) and his mother (II4). However, our patients have not presented any renal abnormalities at present. Patient 1 and 3 whose *FOXC1* genes were deleted did not show renal abnormalities either. Therefore, we suspected *FOXC1* gene is suspected to have some etiological contribution in renal abnormalities but not be a single genetic factor, which is consistent with the conclusion with previous studies [17].

In this study, we reported a patient with developmental delay, recurrent infections, congenital choroidal defects, and craniofacial abnormalities, such as frontal bossing, higharched palate, long philtrum, thin upper lip, abnormal nasal bridge morphology, narrow eyelids, and upper eyelid entropion, suggestive of 6p deletion and 6p duplication syndrome. Since the resolution of the chromosome karyotype is larger than 5 Mb, only a duplication in 6p of our proband was detected. Further molecular analysis helped to more accurately assess a 0.66-Mb telomeric deletion and disclosed a contiguous duplication. In addition, it should be emphasized that the patient’s mother showed a similar phenotype and genotype as her son, which further indicated the pathogenicity of the CNVs. Our observations contribute to the clinical features of contiguous deletion and duplication in 6p. In addition, although the interpretation of genotype–phenotype correlations is not a simple task even in the era of molecular techniques, we still tried to connect the genotype with phenotype. After taking all the evidence into account, we suggested that *FOXC1*, *NRN1*, *SERPINB6, TUBB2A*, *IRF4, and RIPK1* gene may contribute to the phenotype of our patient.

## Supplementary Information

**Additional file 1: Fig. S1**. The cytogenetic and molecular analysis of proband’s parents. **A** CNV-seq result of father; **B** CNV-seq result of mother; **C** Chromosome karyotype of mother

## Data Availability

The datasets used and/or analyzed during the current study are available from the corresponding author on reasonable request.

## Abbreviations

CNVs: Copy number variations
CNV-seq: Copy number variation sequencing
WES: Whole exome sequencing
