# Supplementary figures and images for: Novel maternal duplication of 6p22.3-p25.3 with subtelomeric 6p25.3 deletion: new clinical findings and genotype–phenotype correlations
Source: Mol Cytogenet. 2023 Jun 11;16:11. doi: 10.1186/s13039-023-00640-6 (PMC10259020; doi:10.1186/s13039-023-00640-6)

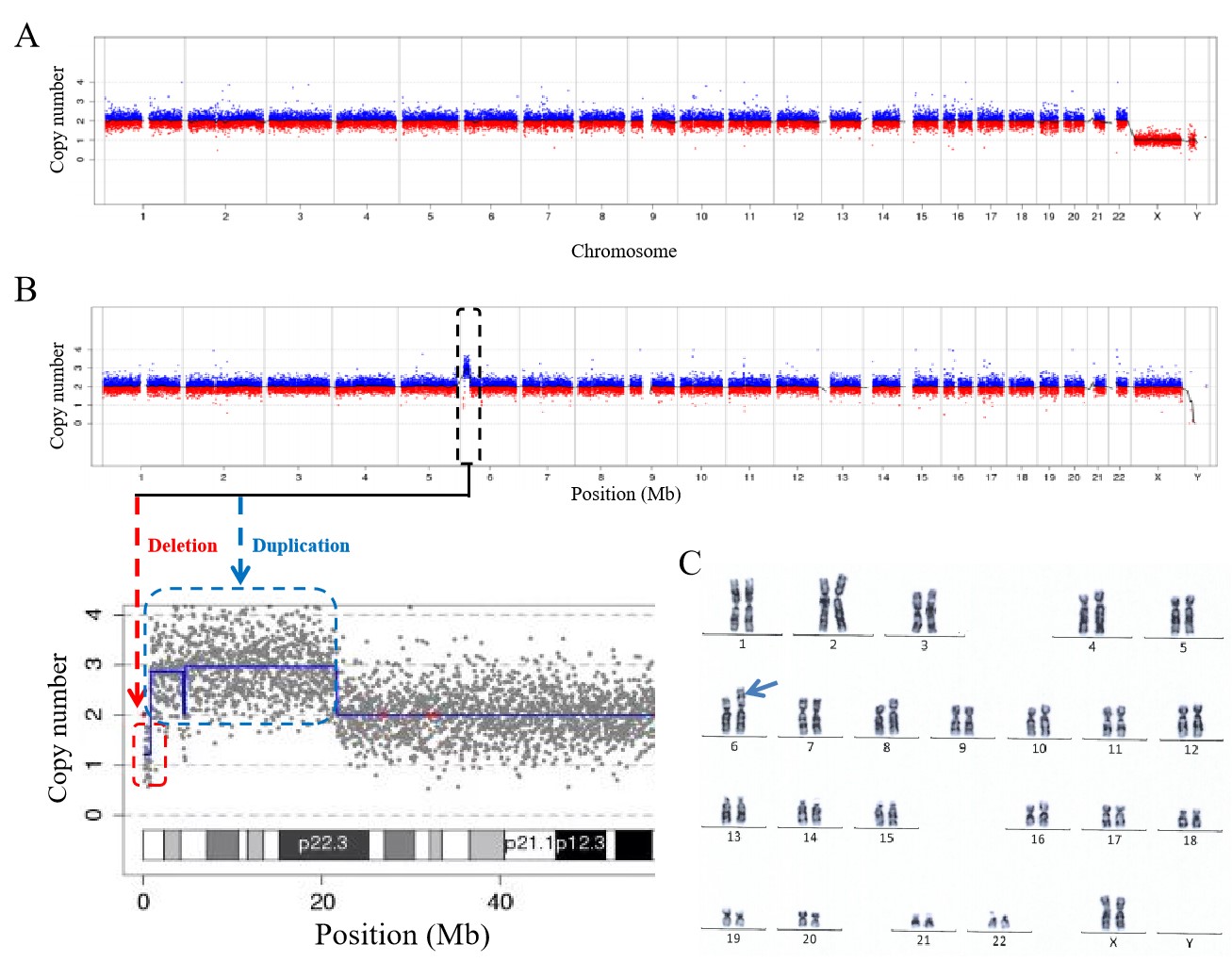

Supplement: Supplementary file 1 — Additional file 1: Fig. S1. The cytogenetic and molecular analysis of proband’s parents. A CNV-seq result of father; B CNV-seq result of mother; C Chromosome karyotype of mother [file 13039_2023_640_MOESM1_ESM.jpg]
